# Early gastric cancer with three gastric gastrointestinal stromal tumors combined with synchronous colon cancer: a case report

**DOI:** 10.1186/s12957-020-02013-4

**Published:** 2020-08-31

**Authors:** Sung Chul Lee, Kwangwoo Nam, Dajeong Nam, Min A. Kwon, Dong-Wook Kim

**Affiliations:** 1 Department of Surgery, Dankook University College of Medicine, 201 Manghyangro, Dongnam-gu, Cheonan, 31116 Chungnam Republic of Korea; 2 Department of Internal Medicine, Dankook University Hospital, Cheonan, Chungnam Republic of Korea; 3 Department of Anesthesiology and Pain Medicine, Dankook University Hospital, Cheonan, Chungnam Republic of Korea

**Keywords:** Colon cancer, Early gastric cancer, Gastrointestinal stromal tumor, Synchronous tumor

## Abstract

**Background:**

There have been very few reports of patients with early gastric cancer (EGC) and colorectal cancer combined with gastric gastrointestinal stromal tumors (GISTs).

**Case presentation:**

We report the case of a patient with multiple tumors that were found at the same time in the abdomen. The patient was a 77-year-old man who was referred for a gastric GIST. Esophagogastroduodenoscopy showed the known lesion (a gastric GIST) on the lesser curvature of the upper body and a new lesion on the lesser curvature of the lower body of the stomach with suspicion of EGC. Computed tomography findings confirmed the presence of a GIST in the stomach and revealed two new lesions. One of these lesions was suspected to be a 4-cm submucosal tumor on the anterior wall of the upper body of the stomach. The other was a wall thickening of the descending colon that demonstrated the possibility of malignancy. Synchronous colon cancer was confirmed on colonoscopy. Laparoscopic near-total gastrectomy with D1+ lymph node dissection and left hemicolectomy were performed sequentially without significant events. The patient was discharged without any postoperative complications.

**Conclusions:**

We reported a rare case of EGC with multiple gastric GISTs combined with synchronous colon cancer.

## Background

The incidence of various primary cancers has increased owing to the increasing age of society and advances in diagnostic imaging technology [1, 2]. In Korea, an organized screening system has significantly improved the prognosis of patients with cancer, especially those with gastrointestinal tract cancer [3]. Synchronous cancers have often been found incidentally during the establishment of a precise diagnosis. The most common synchronous neoplasm is gastric cancer associated with colorectal cancer, and it accounts for 20.1–37.2% of all synchronous cancers [4–6]. There have been numerous reports of simultaneous gastric and colorectal cancers [7–9]. However, there have been very few reports of patients with gastric and colorectal cancer combined with gastric gastrointestinal stromal tumors (GISTs). We present a rare case of early gastric cancer (EGC) with three gastric GISTs combined with synchronous colon cancer. We review the relevant literature and discuss the feasible methods of treatment in such cases.

## Case presentation

The patient was a 77-year-old man who was admitted to our institution due to a gastric GIST detected at a local hospital. The patient first visited the Department of Internal Medicine for an examination. He had no specific complaints other than slight indigestion. The gastric GIST was located on the lesser curvature of the upper body of the stomach. The tumor diameter was approximately 5 cm according to the findings of esophagogastroduodenoscopy (EGD) performed at the local hospital. He had hypertension, which was well controlled with antihypertensive drugs. He had no significant surgical or family history.

**Fig. 1:**
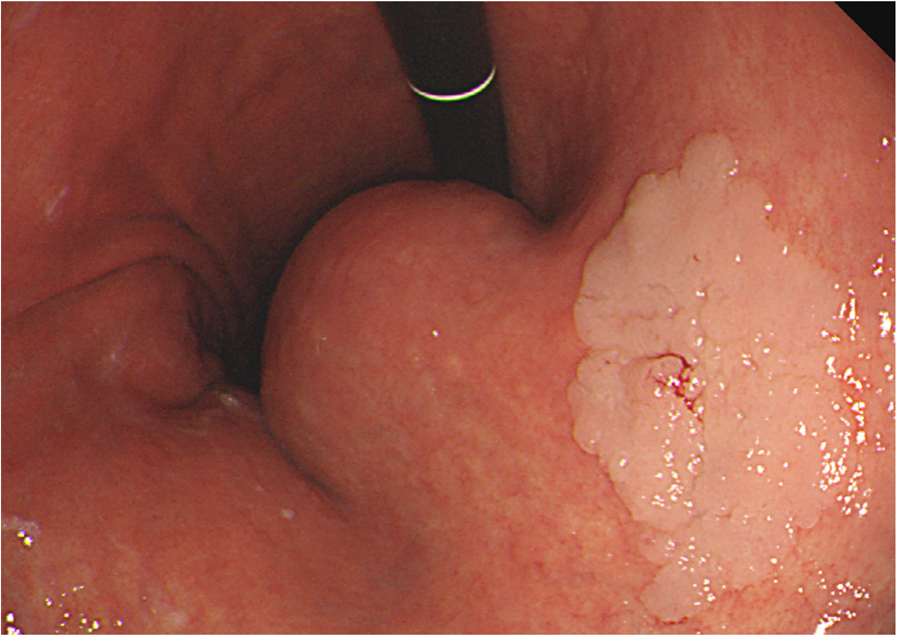
Esophagogastroduodenoscopy showing a submucosal tumor and early gastric cancer

**Fig. 2:**
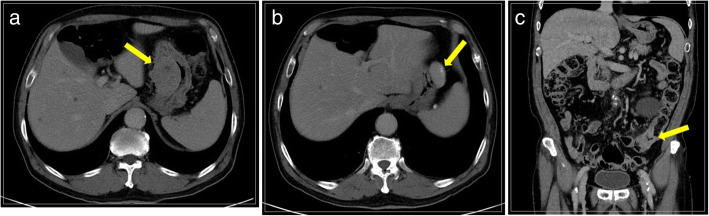
Computed tomography scan showing the presence of two submucosal tumor lesions in the stomach and wall thickening of the descending colon. **a** Mass on the lesser curvature of the stomach. **b** Mass on the anterior wall of the stomach. **c** Wall thickening of the descending colon. Yellow arrow indicates the mass

**Fig. 3:**
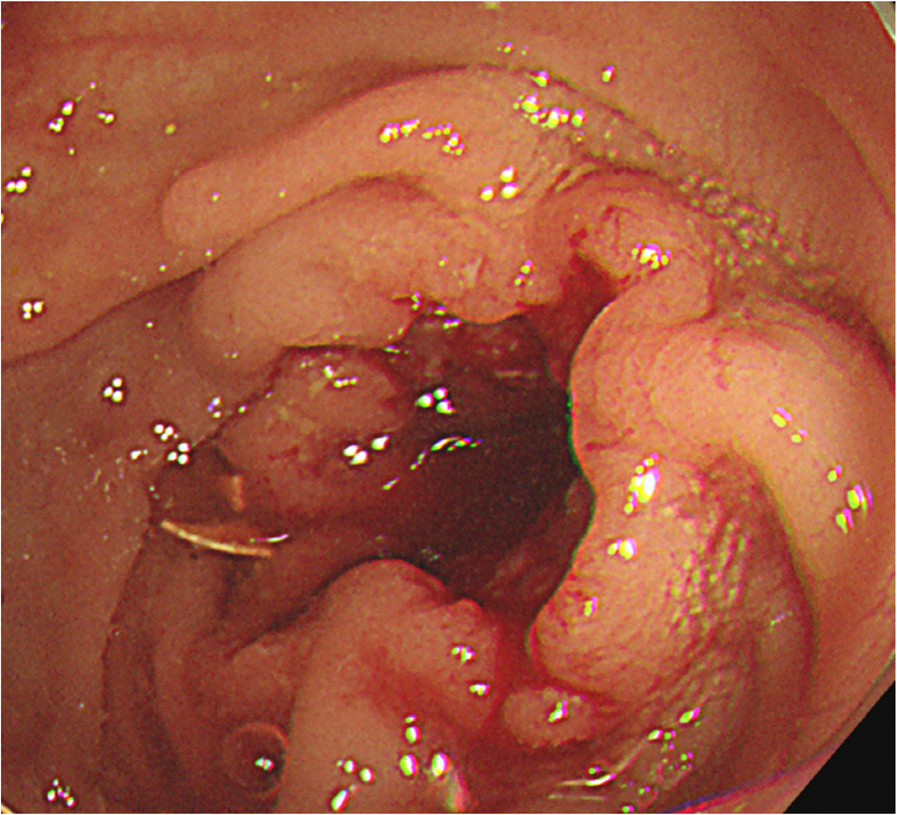
Descending colon cancer was detected via colonoscopy

On physical examination, the patient’s vital signs, including blood pressure, heart rate, body temperature, respiratory rate, and oxygen saturation at room air, were normal. No specific findings were observed on abdominal examination. Another EGD was performed that showed the known lesion (a gastric GIST) on the lesser curvature of the upper body and a new flat-elevated lesion on the lesser curvature of the lower body, leading to a suspicion of EGC (Fig. 1). Computed tomography findings confirmed the presence of a GIST in the stomach (Fig. 2a) and revealed two new lesions. One lesion was suspected to be a 4-cm submucosal tumor on the anterior wall of the upper body of the stomach (Fig. 2b). The other lesion was a wall thickening of the descending colon that demonstrated the possibility of malignancy (Fig. 2c). Upon colonoscopy, a 3-cm ulceroinfiltrative mass was observed from 25 to 28 cm above the anal verge (Fig. 3). Positron emission tomography showed no distant metastasis, including in the liver and both lung fields. Biopsy results revealed two gastric GISTs with spindle cell neoplasm and synchronous gastric and colon cancers that were well-differentiated adenocarcinomas. We decided to perform cooperative laparoscopic surgery. The patient’s hemogram showed a white blood cell count of 11,210/μl and hemoglobin levels of 14.5 g/dl. Blood chemistry findings were as follows: albumin, 4.6 g/dl; creatinine, 0.91 mg/dl; blood urea nitrogen, 10.8 mg/dl; and total bilirubin, 1.12 mg/dl. Serum tumor marker levels were also within normal limits: carcinoembryonic antigen, 2.8 ng/ml, and carbohydrate antigen 19-9, 19.0 U/ml.

**Fig. 4:**
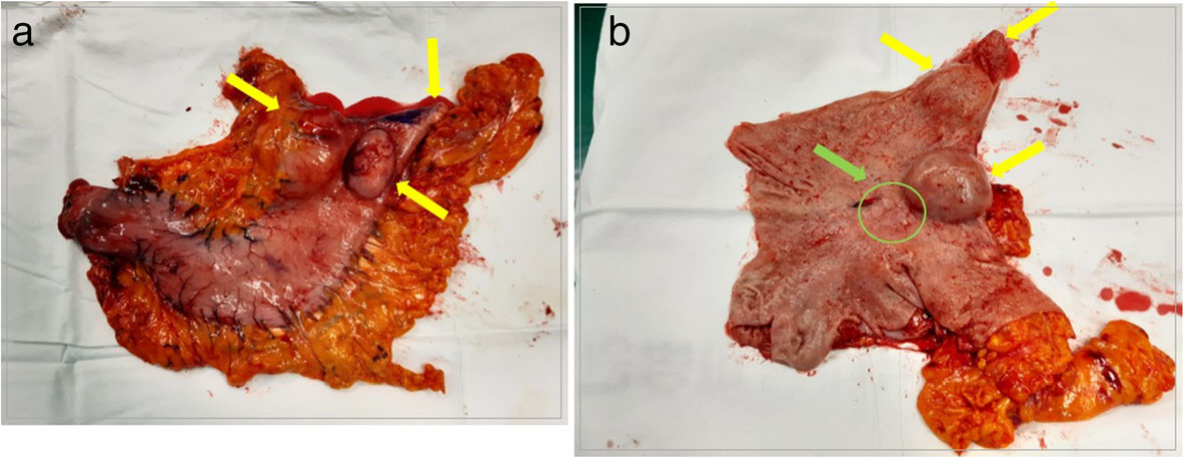
Gross appearance of the resected specimen of the stomach. **a** Original specimen. **b** Specimen opened along the greater curvature (yellow arrows indicate three gastrointestinal stromal tumors; green arrow indicates early gastric cancer)

Laparoscopic surgery was performed according to the standard laparoscopic procedure, and the first 12-mm port was inserted through the umbilicus. For gastrectomy, two operator ports were made on the right upper side of the patient’s abdomen (upper port = 5 mm and lower port = 12 mm). Additional ports were inserted into the right lower (12 mm) and left lower (12 mm) abdominal areas for colectomy. Laparoscopic near-total gastrectomy with D1+ lymph node dissection and left hemicolectomy were performed sequentially without significant events. The stomach and colon were removed through the umbilicus by extending the incision of the umbilical port. In the stomach specimen, we found a 1-cm incidental lesion of a submucosal tumor located on the greater curvature of the high body (Fig. 4). After the surgery, the patient proceeded with sips of water on the second day and began a soft diet on the fourth day. He was discharged 7 days after the surgery without immediate complications.

**Fig. 5:**
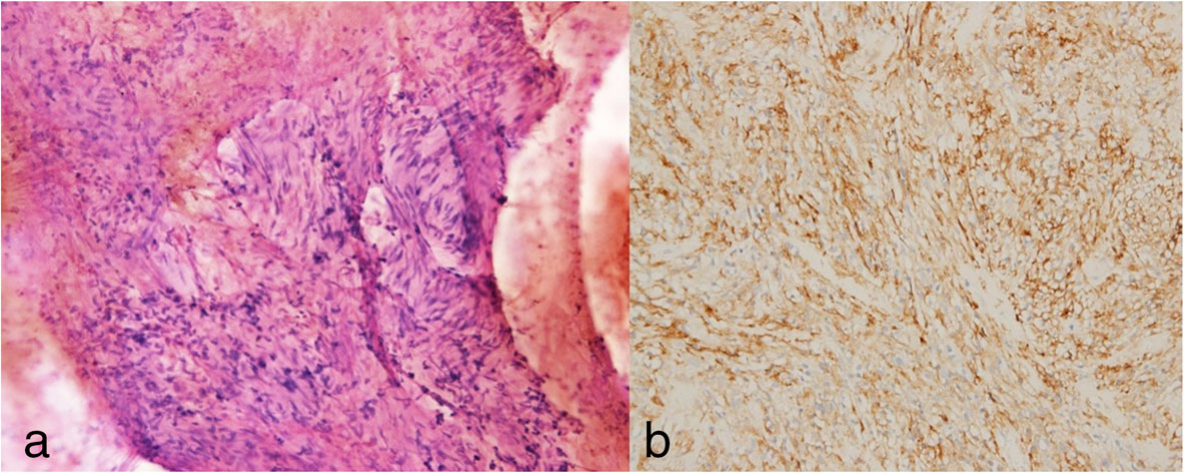
Histopathological examination of the gastric gastrointestinal stromal tumor. **a** Hematoxylin and eosin staining (× 100). **b** c-KIT positivity demonstrated on immunohistochemical staining

Histopathological examination of the EGC revealed a well-differentiated adenocarcinoma with negative resection margins that were staged IA (T1aN0M0) according to the American Joint Committee on Cancer (AJCC) Cancer Staging, 8th edition [10]. Three gastric GISTs (5.5 cm, 4 cm, and 1 cm) posed an intermediate risk as suggested by the modified National Institutes of Health classification system (mitotic index, ≤ 5/50 high-power fields) [11]. Immunohistochemical findings were as follows: c-KIT (+), CD34 (+), DOG-1 (+), S-100 (−), desmin (−), and a low proliferative activity indicated by Ki-67 (Fig. 5). The descending colon lesion was identified as a well-differentiated adenocarcinoma with negative resection margins and was staged IIA (T3N0M0), with low risk, according to the AJCC Cancer Staging 8th edition [10]. No mutations were detected in the *K-ras*, *N-ras*, and *B-raf* genes. In the molecular testing of the colon cancer specimen, tumor cells were confirmed to be diffusely and strongly positive for the epidermal growth factor receptor. Furthermore, the microsatellite instability test showed the status to be stable (microsatellite stable). On the follow-up appointment, the patient was stable without any symptoms and did not require any adjuvant systemic chemotherapy.

## Discussion and conclusions

Synchronous cancer is defined as a simultaneous tumor occurrence in two or more organs or locations. In the gastrointestinal tract, the most common combination is gastric cancer associated with colorectal cancer. Eom et al. [4] reported that colorectal cancer occurring simultaneously with gastric cancer accounts for 20.1% of all synchronous cancers. As a result of the development of diagnostic tools and a well-organized national health insurance system, the detection of gastric cancer, combined with colorectal cancer, has increased in Korea [4, 5].

GIST has been known as a submucosal tumor that can occur anywhere from the esophagus to the anus. The stomach (70%) and small intestine (25%) are the most common locations for its occurrence [12]. GIST is diagnosed via endoscopy or computed tomography, and although the treatment may be slightly different depending on the location or size of the tumor, the treatment of choice is surgical resection. There have been several cases in which gastric GIST was found simultaneously with gastric cancer [13, 14]. Moreover, although several studies have reported on multiple GISTs [15, 16], the exact mechanism of their occurrence remains to be elucidated. However, to the best of our knowledge, the present case is the first reported case in which EGC with three gastric GISTs combined with synchronous colon cancer was detected.

The patient first came to our hospital for further evaluation of a simple gastric GIST. During diagnostic workup, we accidentally detected another gastric GIST, an EGC, and colon cancer. Finally, we detected another GIST in the stomach specimen postoperatively. From the diagnosis to the treatment, various departments collaborated in the present case, i.e., a multidisciplinary approach was organically applied with the involvement of the Departments of Surgery (Division of Gastrointestinal and Colorectal), Gastroenterology, Radiology, Pathology, and Nuclear Medicine. Close consultation and multidisciplinary care are important parts of modern medicine, and these are even more necessary for patients with multiple cancers. Owing to the development of video conferencing and well-designed cellphone applications, it is possible to seek the opinions of other specialists without a face-to-face conversation.

An EGC could be removed via endoscopic submucosal dissection, and GISTs could be treated via wedge resection. However, two of the GISTs in our patient were large and located in the upper body of the stomach. Thus, we were concerned about complications such as a stricture, and we decided to perform total gastrectomy for the stomach lesions. After performing lymph node dissection, we deemed near-total gastrectomy to be feasible, leaving a small proximal part of the stomach without esophageal transection. Consequently, avoiding the serious complication of anastomosis of esophagojejunostomy, the EGC was removed with a sufficient proximal margin, and the GISTs were resected completely. Subsequently, conventional colorectal surgery was performed.

In our patient, all the procedures were laparoscopically performed. A long midline incision would have been inevitable if we could not proceed with the laparoscopic technique for gastrectomy and colectomy. This type of incision could lead to several complications, such as increased postoperative pain, reduced ambulation, and increased length of postoperative hospital stay [17]. Laparoscopic surgery has recently become a popular option and has been gradually replacing the conventional open surgery in several fields of abdominal surgery. The popularization of three-dimensional scopes, development of automatic linear staplers, and improvement in the surgical skills of surgeons are important factors for the recent trends of laparoscopic surgery. For treating EGCs, the laparoscopic approach has become a treatment of choice according to the domestic guideline [18]. Likewise, indications for laparoscopic treatment of colorectal cancer have been gradually expanding [19].

Lifestyle as well as environmental and genetic factors acts synergistically in the pathogenesis of synchronous cancer. Lifestyle factors such as the use of tobacco, alcohol, and nitrosamines influence cancer pathogenesis [20]. Moreover, patients with a history of multiple cancers should undergo a complete evaluation including family history and genetic counseling. Genetic testing is usually performed when multiple synchronous colorectal cancers occur simultaneously at a young age or when concurrent cancers occur in other organs [21]. However, when gastric and colorectal cancers occur simultaneously, both have usually been found to be primary lesions. We could not find any particular risk factors, including genetic mutations, in this case, and there are few specific genetic tests for patients with concurrent cancers [2].

We experienced a rare case of a patient with an EGC with multiple gastric GISTs combined with synchronous colon cancer. In the future, if similar cases are accumulated in multiple studies, it would be necessary to investigate genetic mutation testing for these synchronous cancers.

## Data Availability

All data generated or analyzed during this study are included in this published article.

## Abbreviations

EGC: Early gastric cancer
EGD: Esophagogastroduodenoscopy
GIST: Gastrointestinal stromal tumor
