# Correction to: Early gastric cancer with three gastric gastrointestinal stromal tumors combined with synchronous colon cancer: a case report

**DOI:** 10.1186/s12957-020-02031-2

**Published:** 2020-09-30

**Authors:** Sung Chul Lee, Kwangwoo Nam, Dajeong Nam, Min A Kwon, Dong-Wook Kim

**Affiliations:** 1 Department of Surgery, Dankook University College of Medicine, 201 Manghyangro, Dongnam-gu, Cheonan, 31116 Chungnam Republic of Korea; 2 Department of Internal Medicine, Dankook University Hospital, Cheonan, Chungnam Republic of Korea; 3 Department of Anesthesiology and Pain Medicine, Dankook University Hospital, Cheonan, Chungnam Republic of Korea

**Correction to: World J Surg Onc 18, 231 (2020)**

**https://doi.org/10.1186/s12957-020-02013-4**

Following publication of the original article [1], the authors identified an error in the author name of Min A Kwon.

The incorrect author name is: Min A. Kwon

The correct author name is: Min A Kwon

The author group has been updated above and the original article [1] has been corrected.
